# Water availability affects the relationship between pollen intensity and seed production

**DOI:** 10.1093/aobpla/plab074

**Published:** 2021-12-02

**Authors:** Wilnelia Recart, Diane R Campbell

**Affiliations:** 1 Department of Biology, University of San Diego, 5998 Alcalá Park, San Diego, CA 92110, USA; 2 Department of Ecology and Evolutionary Biology, University of California, Irvine, 321 Steinhaus Hall, Irvine, CA 92697-2525, USA

**Keywords:** *Phacelia parryi*, pollen deposition, pollen intensity, pollen limitation, resource limitation, seed production, water availability

## Abstract

Seed production can be affected by water availability and also depend on the amount (pollen intensity) and quality of pollen deposited. The way pollen receipt on the stigma translates into seeds produced follows that of a saturating dose–response. Not only can water availability and pollen intensity each influence seed production, these factors could interact in their effects on seed production. Changes to the relationship between seed production and pollen intensity can in turn influence pollinator effectiveness and pollinator-mediated selection. We asked how water availability affected indices of plant fitness (seed set, fruit set and seed mass) and the relationship between pollen intensity and seed production in *Phacelia parryi*. We conducted a greenhouse experiment where we manipulated water availability (either high- or low-water) to pollen recipient plants and hand-pollinated each plant with a range of pollen intensities. We conducted 703 hand-pollinations on 30 plants. For each hand-pollinated flower we measured pollen deposited, seed production and seed mass. We then generated a piecewise regression of the relationship between pollen intensity and seed production, and determined average effects of water on plant fitness measures. This experiment was paired with a field observational study aimed to document natural variation in pollen deposition. Average seed production per fruit was 21 % higher in the high-watered plants. The relationship between pollen intensity and seed production differed between the two water treatments. Plants under high-water exhibited a wider range in which pollen deposition increased seed production. Average natural pollen intensities fell within different regions of the piecewise regression for low- and high-water plants. Water availability can alter the efficiency by which pollen received is translated into seeds produced. Our greenhouse data suggest that only under certain pollen intensity environments will water availability affect how pollen received is translated into seeds produced.

## Teaser

Pollen deposition and plant resources (such as nutrient or water availability) limit seed production, yet interactions among these factors have rarely been considered. Using the California native plant, *Phacelia parryi*, scientists showed how the relationship between pollen deposition and seed production is altered by drought. This effect of drought changed the pollen range within which additional pollen deposition increased seed production. They then describe how changes in this pollen-to-seed relationship could be used to know when plants are expected to be pollen-limited, when drought affects pollinator effectiveness, and the impacts of abiotic conditions on seed production.

## Introduction

The transfer of pollen via animal or abiotic vectors, a critical component of sexual reproduction in flowering plants, determines the amount of pollen deposited on a stigma (hereafter referred to as ‘pollen intensity’). Once pollen is deposited onto a stigma, post-pollination processes (such as pollen–style interactions, the quantity and quality of ovules and resources for expanding seeds) determine seed production. Environmental factors experienced by pollen-receiving plants could influence these post-pollination processes and affect seed production. For example, even when pollen intensity remains the same, plant interactions with herbivores and microbes can alter seed production (Hawkes and Sullivan 2001; Thomson *et al.* 2004; Lau and Lennon 2012). In other instances, abiotic conditions—such as water, light and nutrient availability—experienced by the maternal plant affect seed production despite similar pollen intensities (de Jong and Klinkhamer 1989; Campbell and Halama 1993; Galen 2000; Asikainen and Mutikainen 2005; Kilkenny and Galloway 2008). These environmental effects on seed production are usually evaluated using constant levels of either field average or saturating levels of pollen intensity. Because the amount of pollen reaching a stigma varies among individual plants and flowers (Herrera 2004; Arceo-Gomez *et al.* 2016), and environmental factors can influence post-pollination processes, it is possible that pollen intensity interacts with environmental factors to influence seed production. One way environmental factors could interact with pollen intensity is if environmental conditions, such as water availability, affect ovule provisioning. In that case, under drought, pollen intensity might have little effect on seed production, as seed production is limited by water (through effects on ovule provisioning), whereas, when water is abundant, flowers receiving more pollen would have the resources to produce more seeds. Therefore, the potential exists for environmental conditions, such as water availability, to interact with pollen intensity to influence seed production.

The response of seed production to pollen intensity is known to vary across individuals in some populations. For example, *Lesquerella fendleri* shows great intraspecific variation in the pollen-to-seed relationship (Mitchell 1997), although potential environmental causes were not investigated. Another study reported a difference in the relationship between a greenhouse study and a previous study of the same species in natural populations, but did not directly demonstrate the difference was due to the environment (Hildesheim *et al.* 2019). These studies suggest that the relationship between seed production and pollen intensity can be highly variable, but do not ascribe this variation to a particular feature of the environment. In this study, we take the novel approach of examining how an experimental alteration of water availability influences the relationship of seeds to pollen received. Water availability has a high importance to plant reproductive success under natural conditions (Galen 2000; Carroll *et al.* 2001; Waser and Price 2016; Gallagher and Campbell 2017), and is likely to change with future climate change (IPCC 2014; Jiménez Cisneros *et al.* 2014). Thus water availability could be a candidate explaining the variation in the relationship between pollen intensity and seed production.

**Figure 1.**
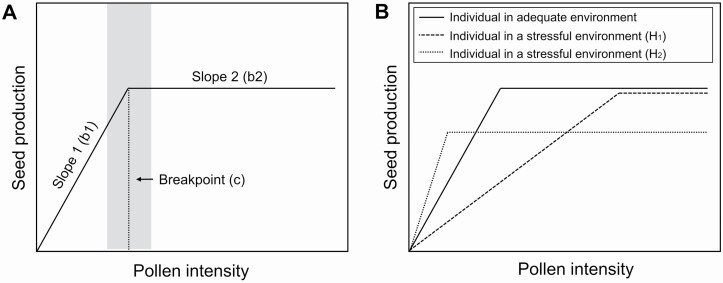
(A) Diagram of pollen-to-seed relationships using piecewise regression analysis. Slope 1 (b1) depicts the most efficient part of the pollen-to-seed relationship and Slope 2 (b2) depicts the least efficient part of the pollen-to-seed relationship. The breakpoint (c) shows the point at which the slope changes. The grey rectangle around the c value represents the c value confidence interval. Figure adapted from Alonso *et al.* (2012). (B) Potential outcomes of how pollen-to-seed relationships could be influenced by stressful environmental conditions. Solid line represents a pollen-to-seed relationship of plants under adequate environmental conditions. Dashed and dotted lines represent potential alternative outcomes of how a stressful environment could influence a pollen-to-seed relationship. Notice that the dashed line is from a pollen-to-seed relationship that exhibits a shallower b1, a later c value and maintains the same intercept for b2. In contrast, the dotted line is from a pollen-to-seed relationship that exhibits a steeper b1, an earlier c value and a decrease in the intercept for b2.

Defining the quantitative relationship between pollen intensity and seed production provides us with a framework to study how the environment influences the fitness gains obtained from different pollen intensities. The relationship between pollen intensity and seed production is generally thought to be a saturating one, in which seed production increases as pollen intensity increases until it plateaus at a saturating level of pollen (Bierzychudek 1981; Haig and Westoby 1988; Ashman *et al.* 2004). One way to quantify the saturating relationship is with a piecewise regression. This method has recently been implemented to document pollen-to-pollen tube relationships (Alonso *et al.* 2012 2013; Arceo-Gómez and Ashman 2014). Here, we adopt it for the pollen-to-seed relationship, condensing it to three attributes: the first slope (b1, see Fig. 1A), when seeds increase most rapidly with greater pollen intensity; the second slope (b2), when seeds increase less rapidly with greater pollen intensity; and the breakpoint (c), defined as the amount of pollen at which the pollen-to-seed relationship switches from high efficiency (b1) to low efficiency (b2) (adapted from Alonso *et al.* 2012). For pollen-to-pollen tube relationships, piecewise regression models have been used to determine differences among species in the degree of limitation by pollen quantity (region before the breakpoint) or by pollen quality (region after the breakpoint) (Alonso *et al.* 2013). These models were also used to describe changes in the degree to which plants are limited by pollen quantity versus pollen quality in female and hermaphroditic individuals found in marginal and central populations (Castilla *et al.* 2016). By using this framework to study the pollen-to-seed relationship, we expect pollen quantity to be most important in the region before the breakpoint and resource limitation experienced by pollen recipients to be most important after the breakpoint. In this framework, aspects of pollen quality or reproductive traits in recipient plants could influence seed production at any point in the pollen-to-seed relationship. Still, after the breakpoint (c) seed production stops being governed by pollen quantity, because additional pollen becomes less important to seed production.

Changes to the pollen-to-seed relationship could influence the degree to which seed production is pollen-limited. For example, if there are changes to the pollen-to-seed relationship due to changes in the maternal environment, then plants under different environmental conditions receiving the same amount of pollen could vary in the degree of pollen limitation. In particular, a plant with a shallow initial slope (dashed line, Fig. 1B) may be more likely to be pollen-limited than a plant with a steep initial slope (solid line, Fig. 1B) when both are receiving naturally low amounts of pollen, and theoretically exhibit the same maximum number of seeds. If environmental conditions decrease the maximum number of seeds, then there is a higher probability of seed production to be resource-limited.

Conceptual models for pollen limitation generally acknowledge that the relationship of seeds to pollen depends upon the environment. For example, Ashman *et al.* (2004) illustrates an increase in both slope and maximum seed set with increasing resources. Understanding how these pollen-to-seed relationships vary with the abiotic environment is important because it indicates how the minimum pollination level needed for full seed set will change. The idea, however, has received little testing. Prior studies have been limited to factorial manipulations of resources with two levels of pollination (Delph 1986; Campbell and Halama 1993; Asikainen and Mutikainen 2005; Sletvold *et al.* 2017) or in one case with the three levels of natural, supplemental pollen and reduced pollen (Brookes *et al.* 2008).

**Figure 2.**
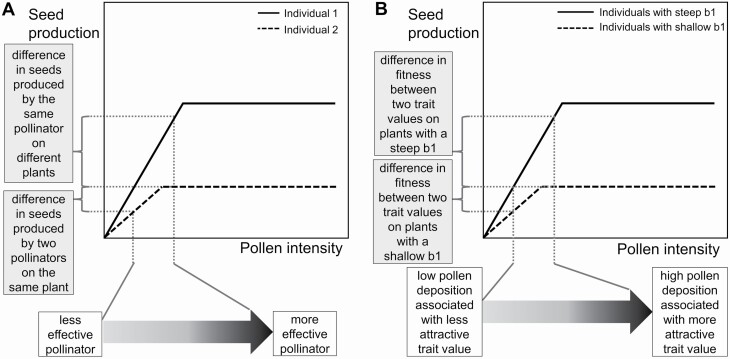
(A) Implications of changes to the pollen-to-seed relationship for pollinator effectiveness: a scenario when two different pollinators visit the same plant (dashed line), and a scenario when the same pollinator species visits plants with different pollen-to-seed relationships (vertical dotted line furthest to the right). Solid and dashed lines represent different individuals. (B) Implications of changes to the pollen-to-seed relationships for pollinator-mediated selection. Solid and dashed lines represent different individuals.

Changes to the pollen-to-seed relationships could also influence pollinator effectiveness, defined as the number of seeds produced from a single pollinator visit compared to that of unvisited flowers (Spears 1983). For example, a shallow initial slope (b1) in a plant can make a visit by two pollinators, one depositing less pollen than the other, contribute more similar amounts to seed production (dashed line, Fig. 2A) and thus have very similar pollinator effectiveness as defined by seeds per visit. In contrast, plants that exhibit differences in their b1 slope (dashed and solid line, Fig. 2A) and are visited by the same pollinator species could display differences in the effectiveness of such pollinator visit (*y*-axis top bracket, Fig. 2A).

A change to the pollen-to-seed relationship can also influence the intensity of pollinator-mediated selection on floral traits through female function (Campbell and Bischoff 2013). If this relationship becomes affected by the environment, then the environment could influence the degree to which pollen deposition affects seed production and, thereby, the intensity of selection. For example, the strength of pollinator-mediated selection could be weakened if b1 is shallow enough that any pollinator preferences would not lead to substantial differences in seed production (Fig. 2B). Alternatively, pollinator-mediated selection could be strengthened if b1 is steep enough that pollinator preferences lead to large differences in seed production (Fig. 2B).

The main goal of this study was to determine if changes in water availability affect the efficiency of translating pollen received to seeds produced. We tested that effect with a greenhouse experiment in which we manipulated water availability and applied known amounts of pollen to stigmas. We then used an observational field study to estimate pollen deposition in a natural population to see whether these values fell in the range over which water availability could influence the impact of pollen deposition on seed production.

## Materials and Methods

### Study species and greenhouse conditions

*Phacelia parryi* (Hydrophyllaceae) is an annual herb native to Southern and Baja California, where it grows in coastal sage scrub and chaparral ecosystems (Bruckman and Campbell 2014). A single plant of *P. parryi* produces from a few to hundreds of flowers. Flowers are hermaphroditic and self-compatible, although flowers produce higher seed production per fruit from outcross pollen (Bruckman and Campbell 2014). Although many species of *Phacelia* have a low ovule number (many species producing 1–10 seeds per flower), a *P. parryi* flower commonly produces from 40 to more than 90 seeds (Walden *et al.* 2013). Plants in natural populations are pollinated by honeybees, bumblebees, solitary bees and some flies (Bruckman and Campbell 2014). *Phacelia parryi* plants bloom from March through May (Walden *et al.* 2013).

*Phacelia parryi* plants were grown from seed during Fall 2014 inside a pollinator-free greenhouse at the University of California, Irvine. Plants were grown in 3-L pots with a soil mixture of 1:1:1 part of peat moss, vermiculite and perlite. Bulk seeds were obtained from the Irvine Ranch Conservancy seed farm. The greenhouse was used for the main component of this study, to determine the influence of water availability on the pollen-to-seed relationship (detailed methods described below).

### Greenhouse water manipulation treatment

Two water availability treatments, low- and high-water, were applied to potted plants germinated from seed in the greenhouse. Water treatment started in October 2014 on plants that had more than two leaves and ended when plants senesced. Every 2 days each low-water plant received 120 mL of fertilized water, and each high-water plant received 120 mL of fertilized water and an additional 120 mL of water filtered through reverse osmosis. Fertilized water contained a mix with 95 % of Peters Professional 20-20-20 complete water-soluble fertilizer and 5 % of Best Ammonium Sulfate 21-0-0 at a concentration of 350–400 ppm in water. The low-water treatment simulated average February precipitation from 1906 to 2014 for Santa Ana, CA, and the high-water treatment simulated twice the average precipitation value (Recart *et al.* 2019). Thirty *P. parryi* individuals—randomly chosen from the germinated bulk seeds—were used as recipient plants, with 15 exposed to a low-water treatment and the other 15 exposed to a high-water treatment. Another 10 plants of *P. parryi* were used as donor plants and received similar water amount as the pollen recipient plants under the high-water treatment, to keep constant effects of water on pollen quality and quantity. To document water treatment effects, soil volumetric water content (VWC) was measured using a soil moisture probe (HydroSense II, Campbell Scientific, Logan, UT, USA)—immediately before watering and 24 h after watering. Plants in the low-water treatment averaged 1.5 % (±1 SE = 0.2 %) and those in the high-water treatment 17.5 % (±1 SE = 0.8 %) VWC, respectively.

### Greenhouse hand-pollination treatments

The biggest purple buds of *P. parryi* recipient plants were emasculated to avoid deposition of self-pollen on hand-pollinated stigmas. Pollen movement from other flowers to emasculated flowers is highly unlikely due to lack of pollinators in the greenhouse and the arrangement of flowers on *P. parryi* plants. Hand outcross pollinations were made after 24 h of emasculation when the stigma was receptive (appeared bifurcated). To ensure that each plant received a similarly wide range of variation in pollen deposition, we performed hand-pollinations in three ways for each plant. To provide a low level of pollen, a toothpick was swabbed from a randomly selected pollen donor, then the toothpick was flicked four times to clear some pollen before swabbing onto the stigma of a recipient flower. For a medium level of pollen, we used the same procedure but flicked the toothpick twice, and for a high level the toothpick was only shaken quickly before swabbing onto the stigma of a recipient flower. Each plant was exposed to all three hand-pollination methods, and each method was replicated at least five times (up to 14 times) on each plant, for a total of 15–42 hand-pollinated flowers per plant. These hand-pollinations represented only a subset of the total number of flowers produced by each *P. parryi* individual. Mean, standard deviations and sample sizes of pollen deposited using each method are provided in **Supporting Information—Table S1**. We did not analyse statistically the effect of the three methods, as the purpose was to ensure a wide enough range of pollen intensity to characterize the form of the relationship between seed production and pollen intensity, which would not be possible if the continuous variable of pollen intensity was collapsed into three levels. A total of 703 hand-pollinations were done. Each hand-pollinated flower was given a unique flower number to be able to relate pollen received to seeds produced.

### Greenhouse stigma collection, pollen count and fitness measurements

Stigmas were collected in a microcentrifuge vial after 48 h of hand-pollination and squashed with basic fuchsin gel on a microscope slide (methods detailed in Kearns and Inouye 1993). Pollen on the stigma slide was counted and related to its flower identification number. Fruits were harvested at the time of ripening, and seeds were counted and weighed to relate seed production to pollen deposition and to calculate average fitness for a plant in terms of average seeds per flower (seed production of all hand-pollinated flowers), fruit set (total number of fruits divided by total number of pollinated flowers) and average seeds per fruit (seed production of hand-pollinated flowers that developed into a fruit). Seeds were separated from fruit tissue and weighed together for each fruit. Seed mass (per seed) was calculated as total seed mass by number of seeds per fruit. We chose to document mean seed production per flower and per fruit, since the former is perhaps the best index of plant fitness, whereas seeds per fruit tests for an impact in expanded fruits only. Note that seeds per flower and seeds per fruit can differ because not all flowers that receive pollen make a fruit, and a threshold amount of pollen is required for a fruit in some species (e.g. Snow 1982).

### *In situ* pollen deposition

We also measured *in situ* pollen deposition in the field, primarily to determine the potential range of pollen deposition values experienced under field conditions. Although previous studies had provided some field data on pollen deposition levels for this species (Bruckman and Campbell 2016), those were for potted plants, not for unmanipulated plants. We used these field data to estimate the average pollen intensity of naturally occurring *P. parryi* plants to determine if it fell in a pollen range where the impact on seed production could depend on water availability, as determined from our greenhouse experiment. For these field plants, we also measured soil moisture to see how soil moisture in the field compared to the greenhouse soil moisture. For each plant, a soil VWC measurement was taken using a 12-cm-long soil moisture probe (HydroSense II, Campbell Scientific, Logan, UT, USA). Stigmas were collected from 31 *P. parryi* individuals flowering at Crystal Cove State Park in Orange County, CA, USA near the Lower Moro Campground (33.575694, −117.794115 WGS 84 Web Mercator) on the side of the trail on a sandy and steep slope in coastal sage scrub habit. At this field site *P. parryi* started blooming between 25–29 March 2018; however, stigmas were collected on 17 April and 23 April 2018 (2 days near the end of the flowering period for this population) when plants were still in bloom. During sampling dates, honeybees and halictid bees were seen visiting *P. parryi* flowers. Stigmas were collected from 31 *P. parryi* flowering individuals, and from each plant we collected one to three stigmas, for a total of 57 collected stigmas from 15 plants on the first date and 16 plants on the second date. The number of open flowers per plant at the time of sampling ranged from 1 to 5; thus one to three collected stigmas accounted for much of the variation each sampled plant experienced on that day. Each stigma was placed in a microcentrifuge vial and squashed with basic fuchsin gel on a microscope slide to allow counting of pollen grains, as described above. Collection date did not influence the average amount of pollen deposited per flower on a plant in an initial ANOVA (*F*_1, 29_ = 0.28, *P* = 0.60).

### Statistical analysis

All statistical analyses were done using the R statistical program version 3.5.2 (R Core Team 2018).

#### Greenhouse data analysis.

We first analysed the overall impact of water availability on four fitness measures averaged by plant identity (regardless of hand-pollination method): seed production per flower (seed production of all hand-pollinated flowers regardless of whether they set fruit or not), proportion of fruits set (total number of fruits divided by total number of pollinated flowers), seed production per fruit (seed production of hand-pollinated flowers that developed into a fruit) and seed mass. The purpose was to provide overall assessments of the impact of water availability as typically performed in studies of plant reproduction. These overall means were analysed using ANOVA with water treatment set as a fixed factor. The same analysis was done to check for absence of a water effect on average pollen deposition per flower (measured as the number of pollen grains counted in the stigma of a hand-pollinated flower) averaged by plant identity. The linear model was implemented using the ‘lm’ function in the ‘stats’ package (R Core Team 2018). Normality of the residuals was tested with the Shapiro–Wilks test using the ‘shapiro.test’ function in the ‘stats’ package (R Core Team 2018).

We then analysed how water treatment affected the pollen-to-seed relationship using two approaches: (i) piecewise regression analyses and (ii) general linear mixed models on raw seed production values per flower. Both approaches used single pollen deposition and seed production values obtained from our hand-pollinated flowers (i.e. 15–42 flowers per plant) and included plant identity as a random effect nested within water treatment. We describe each approach in turn. The piecewise regressions were especially valuable to determine the point at which pollen intensity no longer had much influence on seed production. By using the piecewise regression approach, we were able to distil the curve into two slopes (b1 and b2) with a breakpoint value (c) marking the pollen amount at which the first slope no longer fits the rest of the pollen-to-seed relationship (Alonso *et al.* 2012). As demonstrated by Alonso *et al.* (2012), both a negative exponential model and the piecewise regression approach can produce similar outcomes. We also compared these two approaches with our data and determined that there was no substantial difference between the two models **[see****Supporting Information—Model S1****and****Table S2****]**.

To generate the piecewise regression, we used a linear model where seed number was dependent on plant identity and the amount of pollen deposited. This model was used to estimate b1, b2 and c values (Fig. 1A). This model was run separately for the low-water treatment plants and the high-water treatment plants. The ‘segmented’ function in the ‘segmented’ package was used to run the piecewise regression model. For each model we used the ‘davies.test’ function to determine whether the change in slope was significant (Davies 2002). The ‘slope’ function was used to obtain the slope estimates and associated confidence intervals. The ‘confint’ function was used to obtain the breakpoint estimates and associated confidence intervals.

To test for a significant difference between water treatments in the b1 and b2 slope, for each water treatment, we divided the data into two data sets using the breakpoint value obtained for each water treatment (Arceo-Gómez and Ashman 2014). To detect differences between water treatment in the b1 slope we used all the data points below the breakpoint value (<c), and to detect differences in the b2 slope we used all the data points above the breakpoint value (>c). We then used two linear mixed models to determine if water treatment influenced the b1 and b2 slope, respectively (Arceo-Gómez and Ashman 2014). For these two models (using pollen amount <c and >c, respectively) we used seed number as the response variable and pollen amount and water treatment as crossed fixed effects, with plant identity nested within water treatment and set as a random effect. Including plant identity as a random effect in our models ensured that multiple flowers on the same plant were treated as repeated measures on the experimental unit. An interaction between pollen amount and water treatment would indicate that water availability influenced the slope of seeds on pollen.

A linear mixed model was also used to test whether the second slope differed significantly from zero. Two models were run, one for low-water plants and one for high-water plants. For both models, seed count was set as the response variable, and pollen amount was set as a fixed effect with plant identity set as a random effect. A *t*-test was done to compare the breakpoints between the low- and high-water treatments using their estimates of mean and standard error across plants.

Our second approach to evaluating the change in the pollen-to-seed relationship was to use a general linear mixed model to analyse seed number of individual flowers. Unlike the piecewise regression, this model allows for a smooth relationship between seeds and pollen intensity. We tested for both a linear and quadratic effect of pollen amount on seed number, and whether those relationships were influenced by water treatment (low- versus high-water) as assessed by interactions between water treatment and the linear or quadratic effect of pollen. For this model we tried both a Poisson distribution and a Gaussian distribution, and then settled on a Gaussian which provided a better fit and yielded a much lower Aikake information criterion (AIC) value (AIC = 15 918 and 6526 for Poisson and Gaussian, respectively). In this model we set seed number as the response variable, and included as crossed factors the linear pollen term by water treatment and the quadratic pollen term by water treatment, with plant identity nested within water treatment and set as a random effect.

The general linear mixed models were specified with the ‘glmmadmb’ function of the ‘glmmADMB’ package (Fournier *et al.* 2012; Skaug *et al.* 2016). For these models, we used the ‘Anova’ function in the ‘car’ package, set to type 3 sum of squares to detect effects of our explanatory variables on the response variable (Fox and Weisberg 2011).

#### ***In situ pollen deposition***.

*In situ* pollen deposition data were used to calculate the average pollen deposition experienced by a *P. parryi* plant which we then used to see where field pollen deposition falls in our pollen-to-seed relationship model. We also compared soil moisture in the field to soil moisture experienced by greenhouse plants to see which of our water treatments mimicked more closely field conditions.

## Results

At the whole-plant level, the water treatment primarily influenced average seed production per fruit, which was 21 % higher in the high-water availability than in the low-water availability treatment (*F*_1, 28_ = 6.80, *P* = 0.02; mean ± 1 SE: low-water = 42.09 ± 2.58, high-water = 51.06 ± 2.27). We did not detect an effect of water availability on average fruit set (*F*_1, 28_ = 0.26, *P* = 0.62; mean ± 1 SE: low-water = 0.72 ± 0.04, high-water = 0.69 ± 0.03) or average seed mass (*F*_1, 28_ = 1.54, *P* = 0.23; mean ± 1 SE: low-water = 0.30 ± 0.01, high-water = 0.28 ± 0.01). The effects on fruit set and seeds per fruit together led to an estimated increase of 19 % (although not statistically significant) in average seeds per flower (*F*_1, 28_ = 4.00, *P* = 0.06; mean ± 1 SE: low-water = 29.61 ± 2.04, high-water = 35.32 ± 2.00). Average pollen transferred per flower to each pollen recipient plant did not differ significantly with water treatment (*F*_1, 28_ = 3.69, *P* = 0.07; mean ± 1 SE: low-water = 73.88 ± 1.71, high-water = 84.20 ± 5.09).

**Figure 3.**
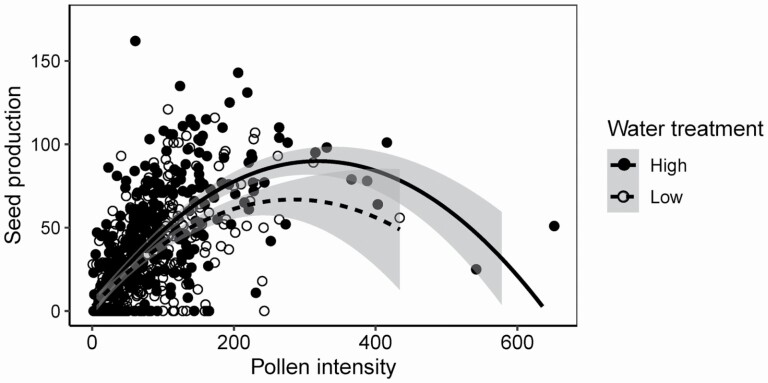
Scatterplot of pollen intensity and seed production data obtained from the 703 hand-pollinations. White circles represent hand-pollinations to low-water treatment plants and black circles represent hand-pollinations to high-water treatment plants. The quadratic relationship between pollen intensity and seed production is shown with a dashed line for low-water treatment plants and a solid line for the high-water treatment plants. Grey shading around each regression line represents 95 % confidence intervals.

**Figure 4.**
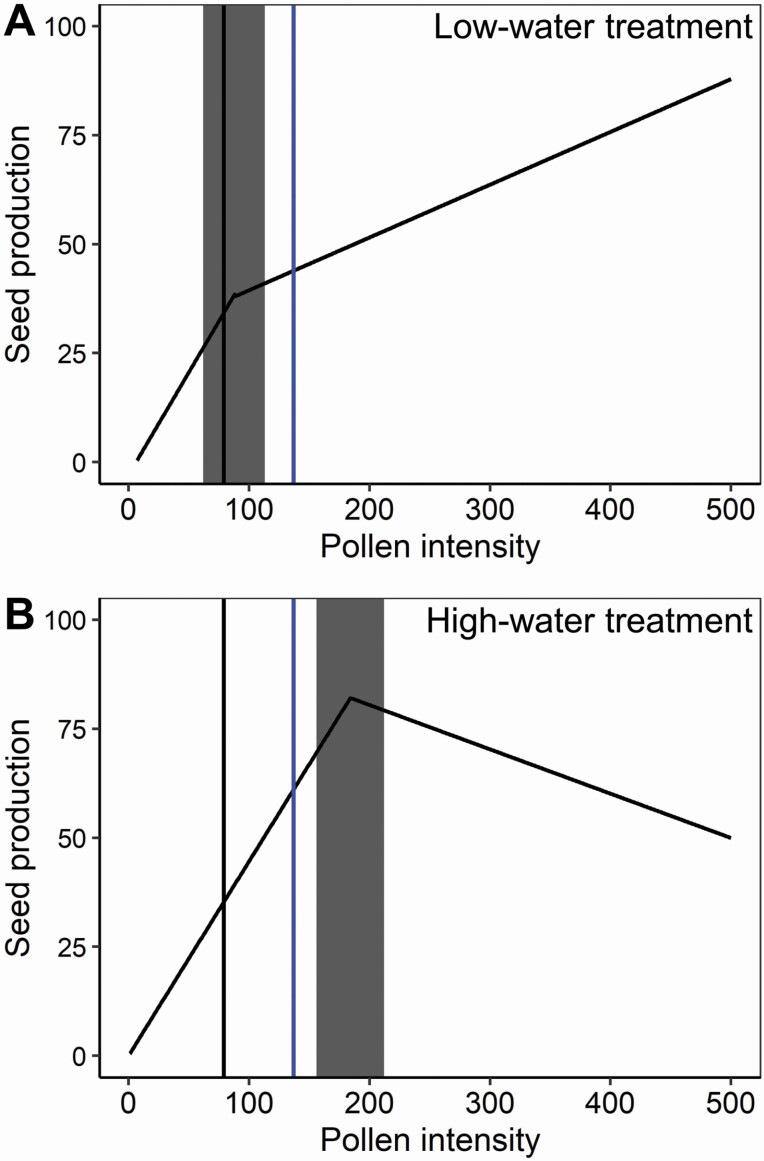
(A) Piecewise regression slopes of pollen recipient plants exposed to the low-water treatment. (B) Piecewise regression slopes of pollen recipient plants exposed to the high-water treatment. For both panels, the grey rectangle represents confidence intervals around the breakpoint. The vertical black solid line represents average *P. parryi* pollen intensity per flower found on *P. parryi* hand-pollinated stigmas. The vertical blue solid line represents average *P. parryi* pollen intensity per flower found on *P. parryi* field-collected stigmas.

**Figure 5.**
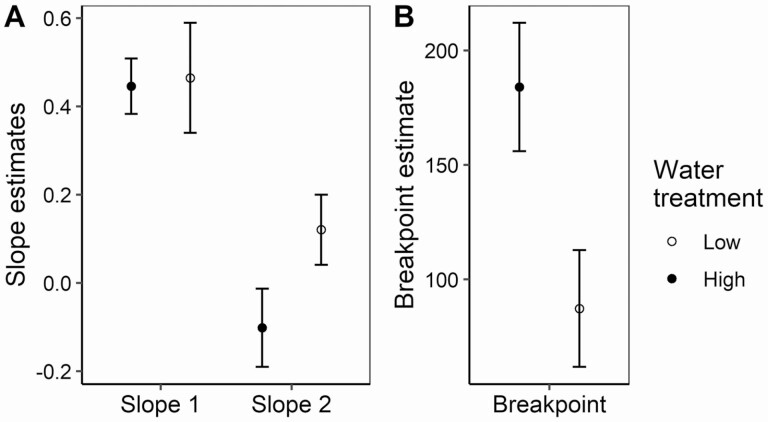
(A) Piecewise regression Slope 1 (b1) and Slope 2 (b2) estimates for pollen recipient plants under low- and high-water treatments. (B) Piecewise regression breakpoint (c) estimates for pollen recipient plants under low- and high-water treatments. Both figure panels show means and 95 % confidence intervals.

Water availability influenced the shape of the relationship between pollen intensity and seeds, as shown by both the piecewise regression approach and the general linear mixed model used to fit a smooth relationship. Overall pollen intensity ranged from 1 to 652 pollen grains across both water treatments (Fig. 3). Overall seed production ranged from 0 to 162 seeds per flower (Fig. 3). In the piecewise regression the b1 slope did not change significantly with water treatment (interaction term: *F*_1, 553_ = 0.05, *P* = 0.81), but the b2 slope did (interaction term: *F*_1, 136_ = 11.29, *P* = 0.001) (Figs 4 and 5A). The b2 slope for the low-water treatment was significantly higher than zero (*F*_1, 107_ = 8.17, *P* = 0.005), whereas the b2 slope for the high-water treatment was not significantly different from zero (*F*_1, 27_ = 3.56, *P* = 0.07). The breakpoint for the two regressions was also significantly different (*t*_1, 699_ = 93.77, *P* < 0.0001), with the low-water treatment having an early breakpoint (mean ± 1 SE: 87.3 ± 13.0 pollen grains) compared to the high-water treatment (mean ± 1 SE: 184.1 ± 14.2 pollen grains; Figs 4 and 5B).

Using the general linear mixed model, we detected an interaction between water availability and the linear effect of pollen amount (Fig. 3; *F*_1, 695_ = 5.73, *P* = 0.02), with pollen recipient plants under low-water conditions having a shallower slope than high-water plants. We detected a negative quadratic effect of pollen amount on seed production (Fig. 3; *F*_1, 695_ = 117.51, *P* < 0.0001). Water treatment and the quadratic effect of pollen amount did not interact to influence seed production (*F*_1, 695_ = 0.09, *P* = 0.77).

In our observational field study, conspecific pollen deposition ranged from 9 to 524 pollen grains and averaged 135.9 (SE = 16.3) grains per stigma. Thus, the mean pollen intensity fell after the breakpoint value for the low-water plants and before the breakpoint value for the high-water plants (Fig. 4). Soil moisture at the time of collection ranged from 0 to 4 % VWC.

## Discussion

Using a continuous range of pollen intensities, we found that water availability affected specific aspects of the pollen-to-seed relationship in *P. parryi.* In a piecewise regression, the breakpoint changed with water as well as the slope after that breakpoint. However, the initial slope at low levels of pollen intensity remained unchanged. In comparison with high-water plants, low-water plants exhibited a smaller range of pollen deposition values over which seed production was limited by pollen quantity (Fig. 4). That change in the breakpoint conforms with the expectation that seed production would be more subject to resource limitation and less so to pollen limitation when water availability is low.

Here we found no influence of water availability on the initial slope (b1) of the seeds to pollen relationship, even though it did change the slope after the breakpoint. In contrast, in other plant species, pollen-to-pollen tube relationships varied within a species in the initial slope depending on whether a plant was at the edge or centre of a population (Castilla *et al.* 2016), and whether or not co-flowering species were present (Arceo-Gómez and Ashman 2014). In those cases, the slope difference likely reflected differences in pollen quality (e.g. via Allee effects, self-pollen or changes in heterospecific pollen), a factor that we constrained to be the same for our two water treatments by using high-water pollen donors. These changes in pollen quality due to changes in abiotic conditions to pollen-producing plants (Turner 1993) could influence the pollen-to-seed relationship. For example, deposition of low-quality pollen could decrease the b1 slope, since additional low-quality pollen may not lead to the same increase in seed production as additional deposition of high-quality pollen. In natural populations of *P. parryi*, it is possible that the initial slope of seeds on pollen would also vary with water availability, because water supplied to donors is known to influence seed production in this species, likely through variation in pollen quality (Recart *et al.* 2019).

Using these seed-to-pollen relationships can help with interpretation of reproductive success in natural populations. For example, if pollen deposition is the same under high- and low-water conditions, then pollen limitation could be high for plants in wet sites or wet years and low for plants under water stress. Here we provide three specific examples of applications to *P. parryi* that illustrate how pollen-to-seed relationships can be used in generating hypotheses on how changes to pollination will affect seed production. First, the average pollen deposition found in naturally occurring *P. parryi* individuals at our field site fell within the initial slope (b1) of high-watered plants but after the breakpoint for low-watered plants (Fig. 4). As a result, changes to pollen intensity could have different effects on seed production depending upon the natural moisture regime. In the specific natural population studied, soil moisture was low at the time of stigma collection (0 to 4 % VWC) and similar to conditions for our low-water treatment greenhouse plants. Thus for that population at that point in time (near the end of its flowering period), assuming that the seed-to-pollen relationships depend only on water availability to pollen-receiving plants and not other factors that could vary between greenhouse and field, we expect small effects of pollen intensity on seed production and greater resource limitation than pollen limitation. It is worth noting that we had only a snapshot of pollen deposition at a particular time point, and in many species pollen deposition is highly variable within a plant, across plants in a population and across populations (Herrera 2002; Burd *et al.* 2009; Alonso *et al.* 2013). Thus, we expect the spatial and temporal context in which a plant blooms to influence the range of pollen deposition experienced by that plant and the effect this pollen deposition has on seed production.

A second application pertains to an earlier study in which the presence of the invasive plant *Brassica nigra* drove variation in pollen deposition to *P. parryi* (Bruckman and Campbell 2016). In that study, pollen deposition on *P. parryi* stigmas was low when *Brassica* individuals were at least 5–7 m away (average of 10 conspecific pollen grains) but high when *Brassica* was within 3 m (average of a hundred conspecific pollen grains) (Bruckman and Campbell 2016). That difference in pollen deposition would lead to a larger difference in seed set for high-water plants than for low-water plants. At 100 pollen grains low-water plants already show a breakpoint in slope beyond which seeds increase little (Fig. 5B). Thus, we predict the distance from a patch of the highly invasive *B. nigra* (Bell and Muller 1973) may have a greater impact on seed set in wet than in drought years, all else equal, as these southern California coastal sage scrub communities swing between precipitation extremes (Kimball *et al.* 2018).

A third application pertains to pollinator effectiveness on a single visit basis. In *P. parryi* pollen deposition by a single visit ranges from an average of 14 pollen grains for non-native honeybees to 28–29 for bumblebees and other native bees (Bruckman and Campbell 2014). All of these values fall well below the breakpoint in the region governed by the initial b1 slope that was not influenced by water treatment. Thus we expect water availability to pollen recipient plants to have little to no impact on relative effectiveness of pollinators on a single visit basis. Thus, native bees would be better pollinators than honeybees in terms of seeds per single visit, as observed by Bruckman and Campbell (2014), regardless of year-to-year differences in water availability.

Here we documented a change in the pollen-to-seed relationships due to changes in water availability, in particular we saw a change in the breakpoint value when flowers switch from being mostly limited by pollen amount to being mostly limited by abiotic resources and pollen quality. Other biotic or abiotic environmental conditions besides water could also have the potential to influence the pollen-to-seed relationship or the pollen-to-pollen tube relationship. For example, co-flowering species in the area influenced the extent to which pollen tube number was limited by pollen quantity in *Mimulus guttatus* (Arceo-Gómez and Ashman 2014). On the other hand, heterospecific pollen deposition on stigmas, and thus possibly also presence of co-flowering species, had little effect on the pollen-to-seed relationship in *Ipomopsis aggregata* (Waser and Fugate 1986), suggesting that the presence of other plant species nearby might not influence the relationship. More studies are needed to identify whether environmental conditions are frequently influencing the pollen-to-seed relationship.

Lastly, environmental factors that have been shown to directly influence seed production can be an ideal starting point to determine whether changes in seed production are caused by changes in the pollen-to-seed relationship. For example, nutrient availability in the soil can directly influence seed production (Campbell and Halama 1993; Asikainen and Mutikainen 2005). Low nutrient availability to pollen recipient plants can decrease pollen germination (Smith-Huerta *et al.* 2008) and could thereby reduce the initial b1 slope describing how seeds increase with pollen. With rapidly changing environmental conditions around the globe (IPCC 2014; Jiménez Cisneros *et al.* 2014), it is important to consider how new temperature, moisture or nutrient regimes might influence the minimum pollination intensity needed for full seed set. Such knowledge could help to predict whether climate change will influence the importance of pollen limitation and guide restoration efforts by indicating whether reproduction in a threatened species could be increased more by managing pollination or resource conditions.

## Conclusion

Water availability can alter the efficiency by which pollen received is translated into seeds produced. In the insect-pollinated *P. parryi*, water availability influenced the breakpoint value at which an increase in pollen receipt no longer has much of an effect on seed production. Moreover, this change in the breakpoint value altered the range of pollen deposition values for which seed production is mainly limited by pollen quantity. Our study suggests that only under certain pollen intensity environments will water availability affect how pollen received is translated into seeds produced.

## Supporting Information

The following additional information is available in the online version of this article—

**Table S1.** Descriptive statistics for pollen deposition obtained from the three methods of hand-pollination and sorted by water treatment to pollen-receiving plants.

**Table S2.** Summary of the negative exponential and piecewise regression models.

**Model S1.** Details and results of the methods used to compare the negative binomial and piecewise regression models used for fitting the relationship of seeds to pollen deposited.

plab074_suppl_Supplementary_Material

plab074_suppl_Supplementary_Data

## Data Availability

An Excel file containing raw data collected for both greenhouse (sheet 1) and field (sheet 2) studies is available as **Supporting Information**.

## Notes

**Natural History and Conservation. Chief Editor**: F. Xavier Picó

## References

[CIT0001] Alonso C , Herrera CM, Ashman TL. 2012. A piece of the puzzle: a method for comparing pollination quality and quantity across multiple species and reproductive events. The New Phytologist 193:532–542. 22007922 10.1111/j.1469-8137.2011.03932.x

[CIT0002] Alonso C , Navarro-Fernández CM, Arceo-Gómez G, Meindl GA, Parra-Tabla V, Ashman TL. 2013. Among-species differences in pollen quality and quantity limitation: implications for endemics in biodiverse hotspots. Annals of Botany 112:1461–1469. 24061490 10.1093/aob/mct213 PMC3806542

[CIT0003] Arceo-Gómez G , Abdala-Roberts L, Jankowiak A, Kohler C, Meindl GA, Navarro-Fernández CM, Parra-Tabla V, Ashman TL, Alonso C. 2016. Patterns of among- and within-species variation in heterospecific pollen receipt: the importance of ecological generalization. American Journal of Botany 103:396–407. 26507115 10.3732/ajb.1500155

[CIT0004] Arceo-Gómez G , Ashman TL. 2014. Patterns of pollen quantity and quality limitation of pre-zygotic reproduction in *Mimulus guttatus* vary with co-flowering community context. Oikos 123:1261–1269.

[CIT0005] Ashman T-L , Knight TM, Steets JA Amarasekare P, Burd M, Campbell DR, Dudash MR, Johnston MO, Mazer SJ, Mitchell RJ, Morgan MT, Wilson WG. 2004. Pollen limitation of plant reproduction: ecological and evolutionary causes and consequences. Ecology 85:2408–2421.

[CIT0006] Asikainen E , Mutikainen P. 2005. Pollen and resource limitation in a gynodioecious species. American Journal of Botany 92:487–494. 21652426 10.3732/ajb.92.3.487

[CIT0007] Bell D , Muller C. 1973. Dominance of California annual grasslands by *Brassica nigra*. The American Midland Naturalist 90:277–299.

[CIT0008] Bierzychudek P . 1981. Pollinator limitation of plant reproductive effort. The American Naturalist 117:838–840.

[CIT0009] Brookes RH , Jesson LK, Burd M. 2008. A test of simultaneous resource and pollen limitation in *Stylidium armeria*. The New Phytologist 179:557–565. 19086296 10.1111/j.1469-8137.2008.02453.x

[CIT0010] Bruckman D , Campbell DR. 2014. Floral neighborhood influences pollinator assemblages and effective pollination in a native plant. Oecologia 176:465–476. 25047026 10.1007/s00442-014-3023-6

[CIT0011] Bruckman D , Campbell DR. 2016. Pollination of a native plant changes with distance and density of invasive plants in a simulated biological invasion. American Journal of Botany 103:1458–1465. 27539258 10.3732/ajb.1600153

[CIT0012] Burd M , Ashman TL, Campbell DR, Dudash MR, Johnston MO, Knight TM, Mazer SJ, Mitchell RJ, Steets JA, Vamosi JC. 2009. Ovule number per flower in a world of unpredictable pollination. American Journal of Botany 96:1159–1167. 21628266 10.3732/ajb.0800183

[CIT0013] Campbell DR , Bischoff M. 2013. Selection for a floral trait is not mediated by pollen receipt even though seed set in the population is pollen-limited. Functional Ecology 27:1117–1125.

[CIT0014] Campbell DR , Halama KJ. 1993. Resource and pollen limitations to lifetime seed production in a natural plant population. Ecology 74:1043–1051.

[CIT0015] Carroll AB , Pallardy SG, Galen C. 2001. Drought stress, plant water status, and floral trait expression in fireweed, *Epilobium angustifolium* (Onagraceae). American Journal of Botany 88:438–446. 11250821

[CIT0016] Castilla AR , Alonso C, Herrera CM. 2016. To be or not to be better pollinated: differences between sex morphs in marginal gynodioecious populations. American Journal of Botany 103:388–395. 26928007 10.3732/ajb.1500167

[CIT0017] de Jong TJ , Klinkhamer PG. 1989. Limiting factors for seed production in *Cynoglossum officinale*. Oecologia 80:167–172. 28313102 10.1007/BF00380146

[CIT0018] Davies RB . 2002. Hypothesis testing when a nuisance parameter is present only under the alternative: linear model case. Biometrika 89:484–489.

[CIT0019] Delph LF . 1986. Factors regulating fruit and seed production in the desert annual *Lesquerella gordonii*. Oecologia 69:471–476. 28311351 10.1007/BF00377071

[CIT0020] Fournier D , Skaug H, Ancheta J Ianelli J, Magnusson A, Maunder M, Nielsen A, Sibert J. 2012. AD Model Builder: using automatic differentiation for statistical inference of highly parameterized complex nonlinear models. Optimization Methods and Software 27:233–249.

[CIT0021] Fox J , Weisberg S. 2011. An {R} companion to applied regression, 2nd edn. Thousand Oaks, CA: SAGE Publications, Inc.

[CIT0022] Galen C . 2000. High and dry: drought stress, sex-allocation trade-offs, and selection on flower size in the alpine wildflower *Polemonium viscosum* (Polemoniaceae). The American Naturalist 156:72–83. 10.1086/303373 10824022

[CIT0023] Gallagher MK , Campbell DR. 2017. Shifts in water availability mediate plant-pollinator interactions. The New Phytologist 215:792–802. 28517023 10.1111/nph.14602

[CIT0024] Haig D , Westoby M. 1988. On limits to seed production. The American Naturalist 131:757–759.

[CIT0025] Hawkes CV , Sullivan JJ. 2001. The impact of herbivory on plants in different resource conditions: a meta-analysis. Ecology 82:2045–2058.

[CIT0026] Herrera CM . 2002. Censusing natural microgametophyte populations: variable spatial mosaics and extreme fine-graininess in winter-flowering *Helleborus foetidus* (Ranunculaceae). American Journal of Botany 89:1570–1578. 21665583 10.3732/ajb.89.10.1570

[CIT0027] Herrera CM . 2004. Distribution ecology of pollen tubes: fine-grained, labile spatial mosaics in southern Spanish Lamiaceae. The New Phytologist 161:473–484. 33873493 10.1111/j.1469-8137.2004.00978.x

[CIT0028] Hildesheim LS , Opedal ØH, Armbruster WS, Pélabon C. 2019. Quantitative and qualitative consequences of reduced pollen loads in a mixed-mating plant. Ecology and Evolution 9:14253–14260. 31938516 10.1002/ece3.5858 PMC6953568

[CIT0029] IPCC . 2014. *Climate change 2014: synthesis report. Contribution of Working Groups I, II and III to the Fifth Assessment Report of the Intergovernmental Panel on Climate Change* (Core Writing Team, RK Pachauri, and LA Meyer, Eds.). Geneva, Switzerland: IPCC.

[CIT0030] Jiménez Cisneros BE , Oki T, Arnell NW, Benito G, Cogley JG, Döll P, Jiang T, Mwakalila SS. 2014. Freshwater resources In: Field CB, Barros VR, Dokken DJ, et al., eds. *Climate change 2014 impacts, adaptation and vulnerability: part A: global and sectoral aspects. Contribution of Working Group II to the Fifth Assessment Report of the Intergovernmental Panel on Climate Change.* Cambridge, UK and New York, NY: Cambridge University Press, 229–269.

[CIT0031] Kearns CA , Inouye DW. 1993. Techniques for pollination biologists. Niwot, CO: University Press of Colorado.

[CIT0032] Kilkenny FF , Galloway LF. 2008. Reproductive success in varying light environments: direct and indirect effects of light on plants and pollinators. Oecologia 155:247–255. 18278516 10.1007/s00442-007-0903-z

[CIT0033] Kimball S , Principe Z, Deutschman S, Strahm S, Huxman T, Lulow M, Balazs K. 2018. Resistance and resilience: ten years of monitoring shrub and prairie communities in Orange County, CA, USA. Ecosphere 9:e02212.

[CIT0034] Lau JA , Lennon JT. 2012. Rapid responses of soil microorganisms improve plant fitness in novel environments. Proceedings of the National Academy of Sciences of the United States of America 109:14058–14062. 22891306 10.1073/pnas.1202319109 PMC3435152

[CIT0035] Mitchell RJ . 1997. Effects of pollination intensity on *Lesquerella fendleri* seed set: variation among plants. Oecologia 109:382–388. 28307535 10.1007/s004420050097

[CIT0036] R Core Team . 2018. R: a language and environment for statistical computing. Vienna, Austria: R Foundation for Statistical Computing. https://www.R-project.org/.

[CIT0037] Recart W , Ottoson B, Campbell DR. 2019. Water influences how seed production responds to conspecific and heterospecific pollen. American Journal of Botany 106:1–9. 31002744 10.1002/ajb2.1273

[CIT0038] Skaug H , Fournier D, Nielsen A, Magnusson A, Bolker B. 2010. glmmADMB: Generalized linear mixed models using ‘AD Model Builder.’ R package. http://glmmadmb.r-forge.r-project.org

[CIT0039] Sletvold N , Tye M, Ågren J. 2017. Resource- and pollinator-mediated selection on floral traits. Functional Ecology 31:135–141.

[CIT0040] Smith-Huerta NL , Carrino-Kyker SR, Huerta AJ. 2008. The effects of maternal and paternal nutrient status on pollen performance in the wildflower *Clarkia unguiculata* Lindley (Onagraceae). The Journal of the Torrey Botanical Society 134:451–457.

[CIT0041] Snow AA . 1982. Pollination intensity and potential seed set in *Passiflora vitifolia*. Oecologia 55:231–237. 28311238 10.1007/BF00384492

[CIT0042] Spears EE Jr . 1983. A direct measure of pollinator effectiveness. Oecologia 57:196–199. 28310176 10.1007/BF00379581

[CIT0043] Thomson VP , Nicotra AB, Cunningham SA. 2004. Herbivory differentially affects male and female reproductive traits of *Cucumis sativus*. Plant Biology 6:621–628. 15375734 10.1055/s-2004-821236

[CIT0044] Turner LB . 1993. The effect of water stress on floral characters, pollination and seed set in white clover (*Trifolium repens* L.). Journal of Experimental Botany 44:1155–1160.

[CIT0045] Walden GK , Patterson R, Garrison LM, Hansen DR. 2013. *Phacelia parryi*. https://ucjeps.berkeley.edu/eflora/eflora_display.php?tid=37536 (30 June 2021).

[CIT0046] Waser NM , Fugate ML. 1986. Pollen precedence and stigma closure: a mechanism of competition for pollination between *Delphinium nelsonii* and *Ipomopsis aggregata*. Oecologia 70:573–577. 28311501 10.1007/BF00379906

[CIT0047] Waser NM , Price MV. 2016. Drought, pollen and nectar availability, and pollination success. Ecology 97:1400–1409. 27459771 10.1890/15-1423.1

