# Supplementary material for: Water availability affects the relationship between pollen intensity and seed production
Source: AoB Plants. 2021 Dec 2;13(6):plab074. doi: 10.1093/aobpla/plab074 (PMC8711293; doi:10.1093/aobpla/plab074)
Supplement: plab074_suppl_Supplementary_Material [file plab074_suppl_supplementary_material.doc]

# **SUPPLEMENTARY MATERIALS**

**Table S1.** Descriptive statistics for pollen deposition obtained from the three methods of hand-pollination (low, medium, or high pollen added) and sorted by water treatment to pollen receiving plants (low or high water).

| **Pollination Method** | **Water Treatment** | **Number of Flowers** | **Mean Pollen Deposition** | **Standard Deviation** | **Standard Error** |
| --- | --- | --- | --- | --- | --- |
| **Low** | Low | 115 | 52.2 | 43.1 | 4.02 |
|  | High | 119 | 63.6 | 77.2 | 7.1 |
| **Medium** | Low | 115 | 74.3 | 51.8 | 4.8 |
|  | High | 120 | 79.4 | 86.0 | 7.9 |
| **High** | Low | 115 | 95.7 | 71.3 | 6.6 |
|  | High | 119 | 107.7 | 80.8 | 7.4 |

**Model S1.** Details and results of the methods used to compare the negative binomial and piecewise regression models used for fitting the pollen amount to the seed production data.

Grouping together data from both low and high-watered pollen-recipient plants, we determine whether the piecewise regression model or the negative binomial model provided a better fit for the data. For the piecewise regression model, we ran a model similar to the one described in the main text but grouping data from both water treatments and not accounting for plant identity. For the negative exponential model, we used the same grouped data and did not account for plant identity. We used non-linear least square analysis to determine the parameter estimates for the negative exponential model using the nls() function in the ‘stats’ package. To compare both models, we calculated the Akaike’s Information Criterion (AIC) for each of the models using the AIC() function in the ‘stats’ package. The smaller the AIC the better the fit, where AIC differences ≤ 2 represent non-significant differences and differences ≥ 2 but ≤ 10 represent nonsubstantially different models (Burnham and Anderson 2004). The difference in AIC values between the two models shows that these two models are not substantially different from each other (Table 1).

**Table S2.** Summary statistics of the negative exponential and piecewise regression models.

| **Model** | **AIC** | **Residual Standard Error (RSE)** | **RSE degrees of freedom** |
| --- | --- | --- | --- |
| **Negative exponential** | 6524.45 | 25.31 | 698 |
| **Piecewise regression** | 6527.45 | 25.35 | 697 |

Burnham KP, Anderson DR. 2004. Multimodel inference: understanding AIC and BIC in model selection. Sociological Methods Research 33: 261–304.
